# Intrinsic brain activity associated with eye gaze during mother–child interaction

**DOI:** 10.1038/s41598-020-76044-y

**Published:** 2020-11-03

**Authors:** Ryo Kuboshita, Takashi X. Fujisawa, Kai Makita, Ryoko Kasaba, Hidehiko Okazawa, Akemi Tomoda

**Affiliations:** 1 Division of Developmental Higher Brain Functions, United Graduate School of Child Development, University of Fukui, Fukui, Japan; 2 Physical Therapy, Department of Rehabilitation, Faculty of Health Science, Fukui Health Science University, Fukui, Japan; 3 Research Center for Child Mental Development, University of Fukui, 23-3 Matsuoka-Shimoaizuki, Eiheiji-cho, Fukui, 910-1193 Japan; 4 Biomedical Imaging Research Center, University of Fukui, Fukui, Japan

**Keywords:** Human behaviour, Social behaviour, Psychology

## Abstract

Mother–child interactions impact child social development and psychological health. This study focused on eye-gaze interactions, especially eye contact as synchronized gaze, which is an important non-verbal communication tool in human interactions. We performed brain-image analysis of mothers and children using resting-state functional magnetic resonance imaging and quantitatively evaluated the quality of mother–child interactions using the Interaction Rating Scale to investigate how it is related to the frequency of mother–child eye contact. As a result, we found a positive correlation between the frequency of eye gaze and the right anterior insula (AI) or middle frontal gyrus in children and a positive correlation with the anterior cingulate cortex (ACC) and precuneus/cuneus in mothers. Especially, when eye contact was made, the association with the right AI in children and ACC in mothers was retained, suggesting the involvement of the salience network responsible for modulating internal and external cognition. In addition, the frequency of eye contact was positively associated with the quality of mother–child interaction. These results suggest that the salience network is a major candidate for the neural basis involved in maintaining efficient eye contact and that it plays an important role in establishing positive mother–child interactions.

## Introduction

The interaction between parents and children constitutes the basis on which children build future relationships and is considered to provide the most influential, important, and meaningful psychological foundations in an individual's life. The attachment style that children establish through interactions with their parents also shapes their social-relationship patterns later in life and can influence psychological health^1^. The parent–child relationship can strongly impact future interactions^2^. Communication through smooth interaction is important for the development of a healthy parent–child relationship, and maternal behavior plays an important role in this process. Although the maternal behavioral repertoire includes gazing at the infant’s face, “motherese” high-pitched vocalizations, facial expressions of positive affect, affectionate touch, and the synchronous adaptation of these behaviors to moments of infant responsiveness^3^, it has been suggested that gaze interaction plays an important role in behavioral synchronization or turn-taking in communication. In fact, humans are extremely sensitive to the directed gaze of others since infancy and recognize its importance as a signal to initiate communication^3^. It has also been noted that eye gaze is importantly implicated in reading the intentions and mental states of others^4^. Thus, eye gaze, which reflects mental states, is essential for mutual understanding in communication, and eye contact is especially considered to constitute the main mode of establishing a communicative context between humans^5^.

Humans tend to use eye contact even in casual communication^6^. Eye contact conveys ample information and is important for establishing human communication^5^; humans receive information from eye contact with their parents since infancy^7^. Through explicit stimuli such as those conveyed via eye contact with the parents, children develop the ability to grasp the intention of the behavioral signifiers^8^. In the development of human relationships, eye contact can provide information, share intentions with each other, regulate parent–child interaction, and promote targeted activities^7,8^. Conversely, it is considered that individuals with autism spectrum disorder (ASD) who have social-communication deficits experience difficulties reacting to and maintaining eye contact^9^. Furthermore, it is believed that children with ASD have difficulty in recognizing the detailed emotions conveyed by the eye gaze of others^10^. The lack of eye contact in ASD could impair smooth communication.

Functional imaging studies have revealed that eye contact can modulate the activity of regions referred to as the social brain network, and a previous review reported the following six typical regions to be implicated: the medial prefrontal cortex (MPFC) and orbitofrontal cortex, posterior superior temporal sulcus (pSTS), anterior insula (AI), fusiform gyrus, and amygdala^8^. Furthermore, recent studies using dual functional magnetic resonance imaging (fMRI) with a hyper-scanning method have revealed that the right AI and anterior cingulate cortex (ACC) were showed pair-specific neural activity related to eye contact during a joint attention task^11,12^. Taken together, task-based functional imaging studies have found that perceived eye contact enhances activation in regions of the social brain network, while this activation interacts with task demands, as well as the social context, to influence precisely which regions in the social brain network are activated^8^. While task-based fMRI has been widely used to explore functional brain regions involved in performing specific tasks, resting-state fMRI has been used to search for intrinsic functional clustering or specialization of brain regions/networks^13^. The intrinsic functional clusters are referred to as resting-state networks (or large-scale brain networks). There are several networks, including the default-mode network (DMN) consisting of the MPFC, pSTS, precuneus/posterior cingulate cortex (CC), as well as the salience network (SN) consisting of the AI and ACC^14^. The two networks (DMN and SN) are considered to be the major resting-state network representatives, and each region in DMN and SN also corresponds to the core regions associated with mentalizing and empathy^14^. Conversely, it has been reported that the intrinsic brain activities at rest are reliably associated with task-induced brain activity and that the regions positively associated with task performance also function as a central executive network (CEN)^15^. Thus, although it is suggested that intrinsic brain functions in the DMN and SN may be involved in human interaction, especially mother–child interaction, via eye contact there has been no direct examination of these relationships.

Moreover, recent reports have examined the availability of intrinsic resting-state brain functions as a potential marker based on the temporal stability within the individual for measuring individual differences in personality traits or the psychiatric status such as the neurodevelopmental phenotypes^16–19^. Although the neurodevelopmental properties that are implicated in eye contact have not been explicitly investigated, it is well known that poor eye contact is an early sign of neurodevelopmental disorders such as ASD^20^, and it has also been reported that resting-state brain functions, such as those in the DMN and SN, are altered in ASD^17,18^. Assaf et al. showed that high-functioning patients with ASD had a DMN similar to that of healthy controls but with reduced function in the MPFC, precuneus, and ACC^17^. Paakki et al. found that adolescents with ASD showed a significant resting-state signal decrease in the right pSTS and AI^18^. In contrast, although eye contact promotes empathy in that it communicates signals that transfer information regarding emotional and mental states^21^, studies with typically developing children have found that resting-state brain activity in the DMN and SN is enhanced in individuals with higher trait empathy^19^. According to a study by Cox et al. on the relationship between affective empathy and resting brain functions using resting-state fMRI (rs-fMRI), there was a significant relationship between empathic behavior and fractional amplitude of low-frequency fluctuation (fALFF) in the insula and temporal pole^19^. Thus, although it is speculated that the individual differences between mother and child in resting brain functions underlying trait empathy and neurodevelopmental phenotype are largely related to the nature of mother–child interactions via eye gaze, especially eye contact, their relevance has not been explicitly examined.

In the current study, we focused on the frequency of gaze interactions, especially of eye contact, by parents and school-aged children engaged in cooperative tasks, and examined the relationship between the frequency of these interactions and the participants’ resting-state brain functions. We selected school-aged children and their mothers as the study population because the network architectures of intrinsic brain functions in children mature after peaking at age 7–9^22,23^ years, while their interactions are frequently maintained before the onset of puberty. In addition, although it is widely known that puberty is the predominant period of mental disorders, the nature of mother–child interaction during primary school age may be involved in later onset. To study the relationship between eye contact and resting-state brain functions, we employed fALFF analysis to explore which resting-state networks might be differentially activated in healthy mothers and children who exhibit higher levels of eye contact compared to those with lower levels of eye contact. Our main hypothesis was that parents and children with higher levels of resting-state brain functions in the DMN and SN would exhibit more frequent eye contact in situations where their coordination to complete a task would be required, and the relevance would be positively involved in the mother–child relationship. Recent intrinsic brain functional analyses have revealed three core resting-state networks (the DMN, SN, and CEN) that are disrupted across many neuropsychiatric disorders, and the relationship between the three core networks and cognitive dysfunction is being investigated^14^. Based on this triple-network model, the current study focused on the intrinsic brain functions in the DMN, SN, and CEN. As the collaborative game task employed in this study required monitoring of the partner’s behavior and/or mental state as well as task execution, it was expected that the DMN would be positively associated and the CEN would be negative associated with eye gaze because over-focusing on tasks interferes with visual attention to partners. Furthermore, it was also expected that SN would be positively involved in achieving effective eye contact at the appropriate timing because the triple-network model suggests that SN modulates dynamic switching between the DMN and CEN.

## Results

### Demographic characteristics

Demographic and clinical characteristics of the study participants are presented in Table 1. The data of 39 pairs of mothers and children were analyzed. No significant difference in demographic data between the sexes was observed in the group of children. Pairs reaching the clinical range in the Beck Depression Inventory second edition (BDI-II) and Autism-Spectrum Quotient (AQ) were not included in the data analysis.

**Table 1:** Demographic data of the participants.

	Children	Mothers
Sex (boys, %)	56.4	–
Age (years, mean ± SD)	8.4 ± 1.3	40.0 ± 4.7
Handedness (right, %)	89.7	100
IQ (mean ± SD)	104.2 ± 14.3	–
BDI-II (mean ± SD)	–	8.1 ± 5.4
AQ (mean ± SD)	17.1 ± 5.8	16.5 ± 6.4
SES (mean ± SD)	–	26.3 ± 13.8

IQ, intelligent quotient; SD, standard deviation; BDI-II, Beck Depression Inventory second edition; AQ, autism spectrum quotient; SES, socioeconomic status.

### Eye-gaze variables (eye gaze and eye contact) and the quality of the mother–child interactions

To assess the degree of behavioral synchronization based on eye gaze in mother–child interactions, we extracted the number of times the participants gazed at each other and the number of eye contacts from the video data while the participants engaged in the game task. In addition, the Interaction Rating Scale (IRS) was used to assess the quality of the mother–child interactions^24^. Table 2 shows the mean and standard deviation of the gaze variables and IRS score and the correlation coefficients between these variables. The average gaze frequency of the child to the mother was 2.4 ± 1.6 (times/min) and that of the mother to the child was 3.4 ± 1.9 (times/min). The average number of eye contacts between the mother and child was 1.2 ± 0.8 (times/min). The average IRS score was 2.4.

**Table 2:** Correlation between the eye-gaze variables and IRS score.

Variables	Mean	SD	Correlations		
			1	2	3
1. Eye-gaze (Children) (times/min)	2.40	1.6			
2. Eye-gaze (Mothers) (times/min)	3.40	1.9	0.473**		
3. Eye-contact (times/min)	1.20	0.8	0.819**	0.708**	
4. IRS score	1.79	0.1	0.285^n.s^	0.344*	0.322*

IRS, Interaction Rating Scale; SD, standard deviation.

* *P* < 0.05; ** *P* < 0.01; NS: not significant.

Regarding the correlations, there was a high positive correlation between eye gaze and eye contact for both the children and mothers (children: r = 0.819, *P* < 0.001; mothers: r = 0.708, *P* < 0.001), and there was also a moderate positive correlation between the maternal and child gaze frequency (r = 0.473, *P* = 0.002). Moreover, a significant positive correlation was observed between the gaze frequency and the IRS score in mothers (r = 0.344, *P* = 0.032) but not in children (r = 0.285, *P* = 0.079). However, there was a significant positive correlation between eye-contact frequency and the IRS score (r = 0.322, *P* = 0.045).

### Resting-state brain function associated with the eye-gaze variables in children

**Figure 1:**
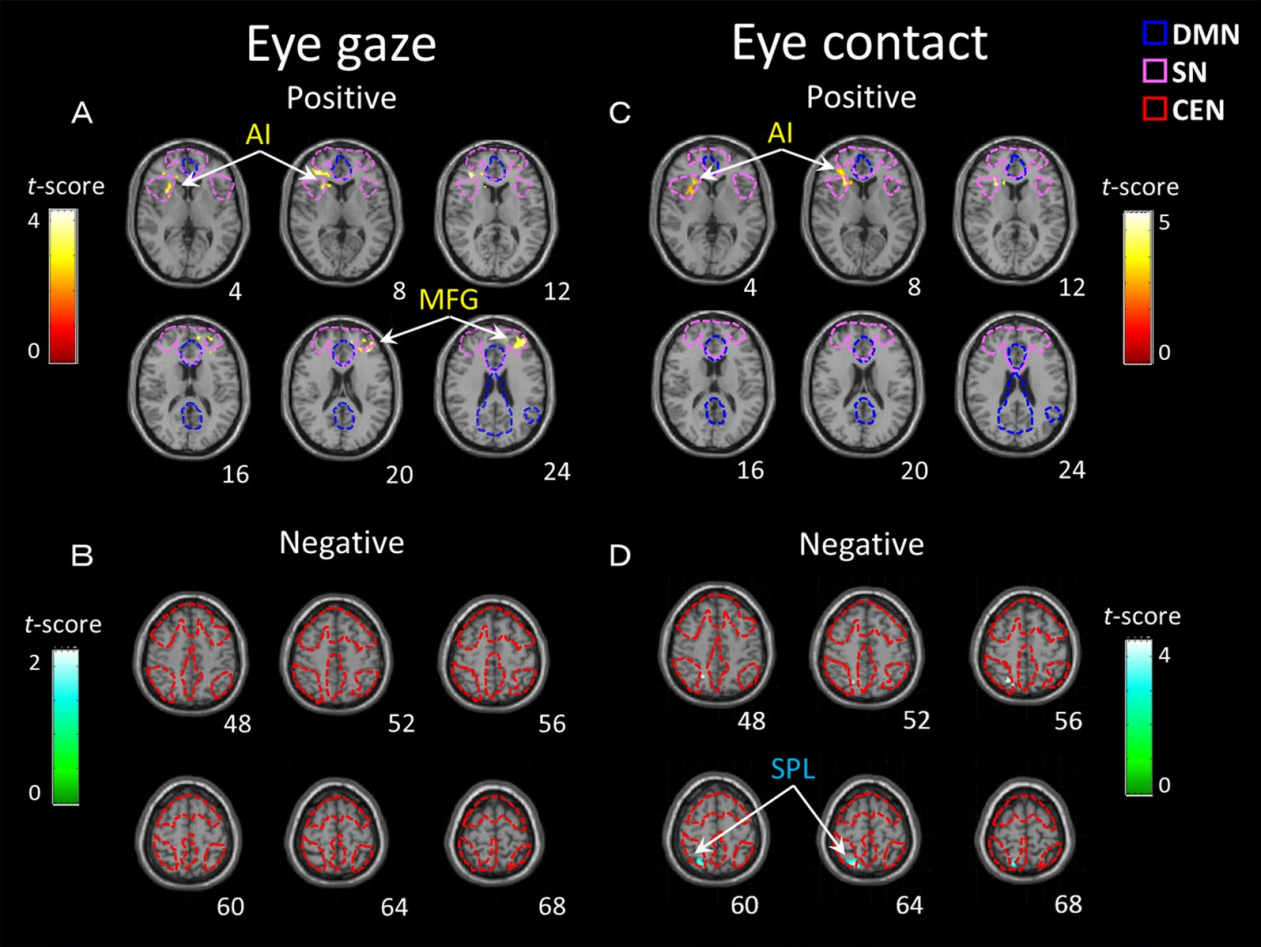
Brain regions in children showing positive correlations (**A**) and negative correlations (**B**)in the eye-gaze conditionand positive correlations (**C**) and negative correlations (**D**) in the eye-contact condition. The statistical threshold for the contrasts was cluster-level *P* < 0.05 family-wise error corrected for multiple comparisons and voxel-level *P* < 0.005 uncorrected for height. The color bar denotes the t-statistic range. The dashed line represents each functional area (DMN, SN and CEN) of the resting network found in previous studies^25–27^. AI: anterior insula, MFG: medial frontal gyrus, SPL: superior parietal lobule, DMN: default mode network, SN: salience network, CEN: central executive network.

The regions implicated in resting-state brain function in children correlated with the eye-gaze frequency variables as shown by the whole-brain analysis presented in Table 3 and Fig. 1^25–27^. In the eye-gaze condition (one-sided gaze toward the mother), a positive correlation was found in the right AI and the left middle frontal gyrus (Fig. 1a) and no negative correlation was found (Fig. 1b). Conversely, under the eye-contact condition (eye contact with the mother), a positive correlation was also found in the right AI but not in the middle frontal gyrus (Fig. 1c), and a negative correlation was found in the superior parietal lobule (Fig. 1d).

**Table 3:** Brain regions showing correlations between the eye-gaze variables in children.

Region (Brodmann area)	Network	Side	MNI coordinates (mm)			*t*-score	Cluster size	Cluster *p*
			X	Y	Z		*kE* (voxels)	(FWE)
**Eye-gaze**								
*Positive correlation*								
Anterior insula (13/14)	SN	R	26	20	10	4.54	333	0.003
Middle frontal gyrus (9)	DMN	L	− 34	40	20	4.26	311	0.005
*Negative correlation*								
None								
**Eye-contact**								
*Positive correlation*								
Anterior insula (13/14)	SN	R	26	24	8	5.69	266	0.012
*Negative correlation*								
Superior parietal lobule (7)	CEN	R	20	− 66	64	4.51	265	0.013

MNI, Montreal Neurological Institute; FWE, family-wise error; SN, salience network; DMN, default mode network; CEN, central executive network.

### Resting-state brain function associated with the eye-gaze variables in mothers

**Figure 2:**
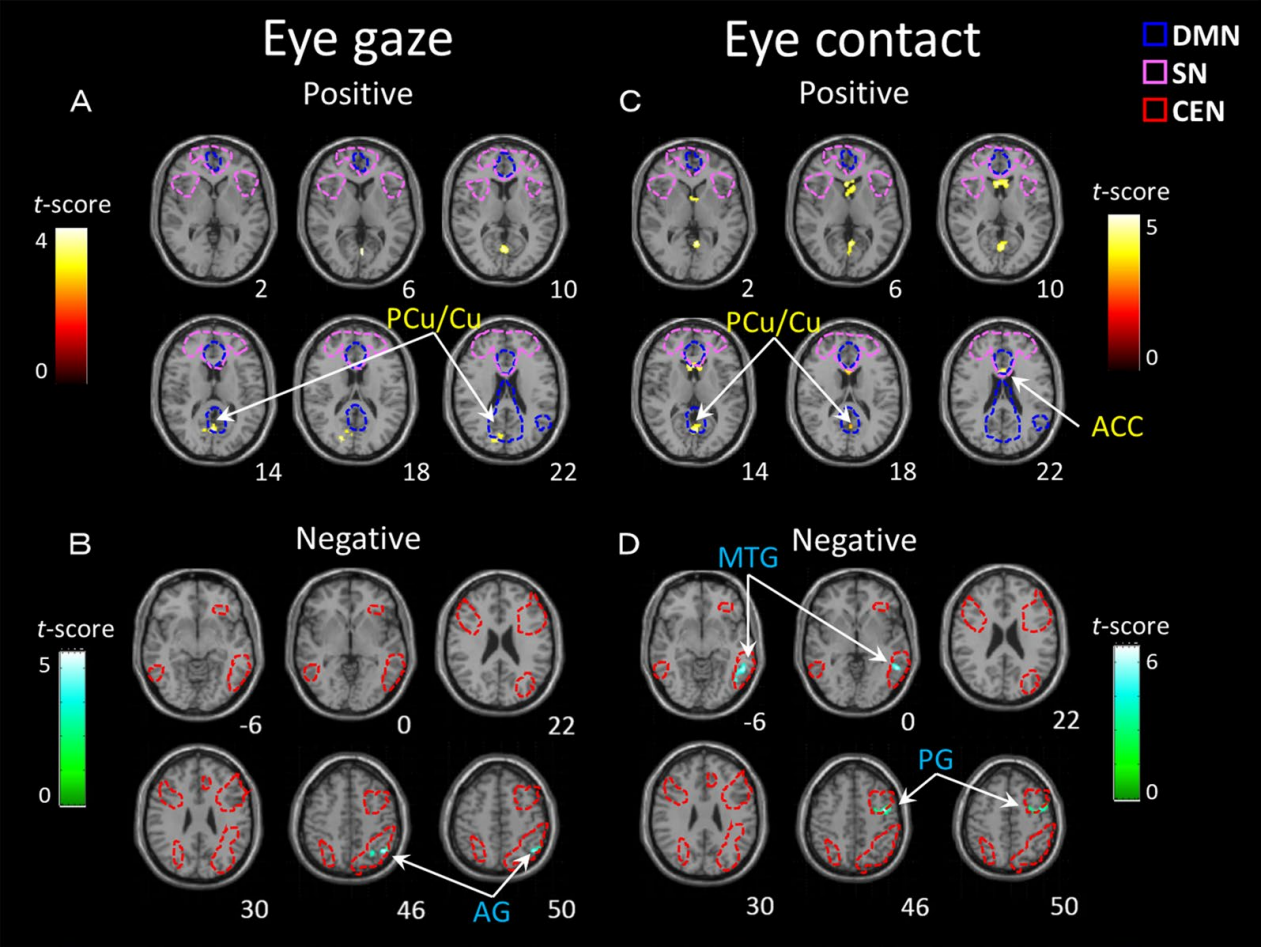
Brain regions in mothers showing positive correlations (**A**) and negative correlations (**B**) in the eye-gaze condition and positive correlations (**C**) and negative correlations (**D**) in the eye-contact condition. The statistical threshold for the contrasts was cluster-level *P* < 0.05 family-wise error corrected for multiple comparisons and voxel-level *P* < 0.005 uncorrected for height. The color bar denotes the t-statistic range. The dashed line represents each functional area (DMN, SN and CEN) of the resting network found in previous studies^25–27^. ACC: anterior cingulate cortex, AG: angular gyrus, Cu: cuneus, MTG: middle temporal gyrus, PCu: precuneus, PG: paracentral gyrus, DMN: default mode network, SN: salience network, CEN: central executive network.

The local regions of resting-state brain function in mothers correlated with the eye-gaze frequency variables as shown by the whole-brain analysis presented in Table 4 and Fig. 2^25–27^. In the eye-gaze condition (one-sided gaze toward the child), a positive correlation was found in the precuneus/cuneus (Fig. 2a) and a negative correlation was found in the left angular gyrus (Fig. 2b). Moreover, under the eye-contact condition (eye contact with the child), a positive correlation was also found in the precuneus/cuneus and ACC (Fig. 2c). A negative correlation was found in the left precentral gyrus and middle temporal gyrus (Fig. 2d).

**Table 4:** Brain regions showing correlations between the eye-gaze variables in mothers.

Region (Brodmann area)	Network	Side	MNI coordinates (mm)			*t*-score	Cluster size	Cluster *p*
			X	Y	Z		*kE* (voxels)	(FWE)
**Eye-gaze**								
*Positive correlation*								
Precuneus/cuneus (7)	DMN		2	− 68	12	3.37	219	0.015
*Negative correlation*								
Angular gyrus (39)	CEN	L	− 28	− 60	46	4.19	279	0.003
**Eye-contact**								
*Positive correlation*								
Anterior cingulate cortex (24)	SN		− 2	16	22	4.04	314	0.001
Precuneus/cuneus (7)	DMN		− 2	− 66	12	4.14	214	0.017
*Negative correlation*								
Precentral gyrus (4)	CEN	L	− 40	− 4	50	6.75	275	0.003
Middle temporal gyrus (21)	CEN	L	− 56	− 50	− 6	4.64	398	0.000

MNI, Montreal Neurological Institute; FWE, family-wise error; DMN, default mode network; CEN, central executive network; SN, salience network.

## Discussion

This study investigated how frequently mothers and children gazed at each other during a cooperative game task, especially how frequently they made eye contact, and examined the relationship of these frequencies with the participants resting-state brain activities. In addition, we also investigated the relationship between eye gaze and the quality of mother–child interactions using the IRS. As a result, we found a positive correlation between the eye-gaze frequency variables and the right AI or middle frontal gyrus and a negative correlation with the right superior parietal lobule in the resting-state brain activity of children. In mothers, we found a positive correlation between the eye-gaze frequency variables and the ACC and precuneus and a negative correlation with the left angular, middle temporal, and paracentral gyri. Especially, when eye contact was made, it was also revealed that the SN (right AI and ACC) was significantly implicated in both mothers and children. In addition, the frequency of eye contact was positively associated with the quality of mother–child interaction. Taken together, these results suggest that eye contact plays an important role in the quality of mother–child interactions and that the SN is a major candidate for the neural basis involved in maintaining efficient eye contact.

In the resting-state brain function of children, we found that the right AI and the medial frontal gyrus (MFG) were positively associated with the frequency of gaze, and that the right AI was particularly involved in achieving eye contact. The function of the right AI during the resting state is central to the SN responding to a wide range of stimuli related to emotional and motivational states^28^. In particular, the right AI is a critical component of the SN, which mediates interactions between large-scale brain networks involved in the externally oriented attention by the CEN and internally oriented cognition by the DMN^29^. Furthermore, the right AI is located at the interface of brain cognition, homeostatic, and affective systems and is responsible for the functional connection between stimulus-driven processing and brain regions involved in monitoring the internal milieu^30^. The right AI as part of the SN facilitates the integration of sensory, emotional, and cognitive information for optimal communication, social behavior, and self-awareness^31^. Considering the function of the right AI as part of the SN, it may be the case that children who frequently gaze at their mother can properly switch their attention not only to perform the game task but also to observe the mother’s emotional reaction to the task. In addition, it is also suggested that the children recognize the emotional alterations of the mothers when performing the task and gaze at their mother to share the experience. We also found that the superior parietal lobule, included in the CEN, was negatively involved in eye contact. The superior parietal lobule is involved in executive function^32^ and was found to be activated when focusing attention on attentive tracking^33^. This higher fluctuation in the superior parietal lobule as part of the CEN may induce over-focusing on the task and relatively reduce eye contact with the mother or interest for the surroundings. Regarding the quality of the mother–child interaction, the IRS score was positively associated with eye contact, while no significant correlation was found with eye gaze. This finding suggests that the timing of the eye contact with the mother plays a more important role in the quality of the mother–child interaction than simply gazing at the mother. Moreover, the right AI as part of the SN may contribute to a further extent than the middle frontal gyrus as part of the DMN in attentional switching at the appropriate timing in the interaction.

In the resting brain function of mothers, we found the precuneus/cuneus as functional regions positively associated with eye gaze, and additionally found that the ACC was implicated in eye contact. Although the precuneus is known as a core region of the DMN^34^, the DMN is considered to be involved in mentalizing or Theory of Mind, i.e., the ability to understand and manipulate the psychological states of others^35^. The precuneus was reported to be bilaterally activated during judgements requiring empathy in a functional imaging study^36^. Therefore, the positive relationship between the frequency of gazing at the child and precuneus/cuneus fluctuation may reflect that such mothers are more sensitive to changes in the mental state of children and in the task status during the interaction. In addition, the ACC, which is reportedly a core area of the SN^37^ and shows the highest activity in response to changes in circumstances where there is no prior information^38^, was specifically involved in eye contact. In addition, structural and functional abnormalities in the SN including the AI and ACC have been reported to show abnormal DMN function^39^, and the integrity of the SN is also necessary for the efficiency of DMN activities^31^. Considering the function of the ACC as part of the SN and the relationship between eye contact and resting brain function found in children, higher fluctuation in the ACC may reflect the efficiency of switching between performing game tasks and monitoring the mental state of children. It may be considered that such mothers are able to control their own emotions at the same time as performing tasks and are capable of fruitful collaborative work with their children. Conversely, the left angular gyrus was negatively associated with eye gaze and the left precentral and middle temporal gyri were negatively associated with eye contact. The angular gyrus has a wide range of functional relationships with many brain regions and is called a connection hub because of its functionality^40^. Similarly, the precentral gyrus was suggested to be involved in executing voluntary motor movements^41^ and the middle temporal gyrus to be involved in actions and word meaning processing^42^. As these areas are constituents of the CEN^41,42^, they may have induced over-focus on tasks and interference with eye contact. In relation to the quality of the mother–child interaction, the IRS score was positively associated with both eye gaze and eye contact in mothers, although it was only associated with eye contact in children. This finding suggests that the feasibility of eye contact during mother–child interactions may be highly dependent on the number of gazes from the mothers to the children. Additionally, the coordination and integration of the precuneus/cuneus as part of the DMN and of the ACC as part of the SN may contribute to the quality of mother–child interaction by mediating maternal gaze with more appropriate timing.

In resting brain function, the AI in children and the ACC in mothers were observed as functional regions positively associated with eye contact, and it is noteworthy that both are core regions of the SN. It has been suggested that the AI–ACC-centered SN initiates key control signals in response to salient stimuli or events^32^, where the SN performs an important role in the dynamic switching between the CEN and DMN^43^. In addition, the AI and ACC are also implicated in the functional network related to empathy^44^. Leibenluft et al. reported activation of the AI and ACC as brain regions showing emotional responses when parents saw familiar faces such as those of their own children^45^. Moreover, Carlson et al. stated that children can develop empathy and prosocial behavior by appropriately controlling executive functions^46^. This finding suggests that the function of the SN in the dynamic switching between the CEN and DMN is an important factor in the development of empathy and prosocial behavior during childhood. According to Feldman, the development of empathy in children requires sensitive parental care, especially active involvement and mutual understanding with parent–infant coordinated eye gazing, affective expression, posture, and social communication being important^47^. Thus, positive mother–child interaction using eye contact is also considered an important factor for fostering empathy in children^47–49^, and then in order to build a positive parent–child relationship, eye contact appropriate for the situation may be essential. The participants appeared to consciously employ eye contact to recognize change in the emotional response of the partner and share the experience of the task that required cooperation. This suggested that an important factor to establish a positive interaction is to maintain balance between the CEN and DMN by effectively utilizing the dynamic switching function of the SN.

It is interesting to examine why the functional areas denoting positive involvement of the SN and DMN during eye contact differed between mothers and children. In this regard, although the detailed mechanism is unclear, there may be two possible explanations: neurodevelopmental factors and asymmetrical relationships implicated in the mother–child interaction. According to the former explanation, it has been reported that adults have higher intrinsic resting brain function of the precuneus in the DMN^50^, while children showed stronger activation in the right AI compared to adults during task execution^51^. In addition, DMN involvement in the SN is increasing in adults, while network integration and segmentation between networks is progressing^29^. Taken together, these neurodevelopmental differences in the relationship between functional areas and networks between adults and children may have induced the involvement of the AI in children and of the ACC and precuneus in adults during eye contact. According to the latter explanation, the AI receives convergent multisensory inputs and affective and motivational signals, while the ACC plays a more dominant role in response selection^31^. Therefore, in the performance of collaborative tasks via eye contact, the integration of external information (tasks and mother’s status) and motivation for the task in the right AI was positively involved in children, whereas monitoring the child's mental state based on mentalizing and contextual response selection in the precuneus and ACC may play a significant role in mothers.

Several limitations of the present study should be noted and taken into consideration in future studies. First, this study included a relatively small sample of participants and utilized a cross-sectional design that precluded the identification of causal links between the development of interpersonal relationships, eye contact, and brain functions as their neural basis. Although we approached with a cross-sectional design based on previous studies that evaluated the relationship between resting brain function and behavioral assessment in mother–child interactions^52–54^, it should be stated that the relationship between the participant measurements is solely grounded on correlations. Therefore, their interpretability is limited by possible reverse causality and influence of other variables. In regard to the small sample size, we used a strict approach (family-wise error) rather than the false discovery rate to detect the brain regions showing positive correlations in the both eye-gaze and eye-contact conditions. Longitudinal studies utilizing larger sample sizes are required to more fully elucidate the association between brain development and eye contact underlying positive interpersonal relationships. Second, the frequency of eye contact and observer rating were only used in the assessment of mother–child interactions. In particular, although the IRS was used to assess the quality of the mother–child relationship in this study, the authors themselves were involved in the assessment because it was difficult to involve a third party who would be blind to the research hypothesis because assessing the IRS requires training. It should also be clarified in the future how different modalities, such as duration of eye contact, voice communication, and physical contact, and the quality of the mother–child relationship based on more objective scoring are related to brain function. Third, rs-fMRI was used as a means to visualize the brain function involved in the eye contact between the two persons via interaction tasks. Therefore, although the relationship between resting-state brain function and eye contact was clarified for each individual, it is unclear whether the functional areas identified in this study are involved in eye contact itself. Despite these limitations, this study sheds light on the importance of the relationship of eye contact in mother–child interaction and its neural basis.

In conclusion, we identified DMN areas, such as the MFG and ACC, and SN areas, such as the AI and ACC, in both mothers and children as resting-state brain function-related areas positively involved in gaze in mother–child interaction, but only the SN was involved in eye contact. These results suggest that the participants frequently gazing at their partner can properly switch their attention to both perform the task and observe the partner’s reaction or their status in relation to the task. Furthermore, the frequency of eye contact was positively associated with the quality of the mother–child interaction. This finding suggests that the timing of the eye contact with the mother plays a more important role in the quality of the mother–child interaction than simply gazing at the mother, and then the SN may contribute to attention switching at the appropriate time during the interactions. In the future, it is important to clarify the developmental transition by investigating the parent–child interaction and its neural basis during the adolescent detachment period.

## Methods

### Participants

Forty-six children aged 6–11 years (26 boys, 20 girls; mean age: 8.5 ± 1.3 years) and their mothers (mean age: 40.2 ± 4.5 years; age range: 31–50 years), who were recruited from the local community via advertisements, participated in the present study. All participants were Japanese. The handedness of all participants was assessed using the Edinburgh Handedness Inventory, and their socioeconomic status was assessed using the Hollingshead Index of Social Position as a composite measure^55^. Exclusion criteria for all participants were any contraindications for MRI, physical problems, psychopathology, any history of psychiatric disorders, severe head injury, neurological abnormalities (e.g., epilepsy and tics), and excessive head motion (> 3.0 mm, 3.0 degrees, and mean frame-wise displacement[FD] < 0.5 mm) during the scanning. The participants had no history of exposure to drug abuse, traumatic events, and fetal drug exposure that might have influenced brain development. All participants were right-handed and unmedicated.

The children were confirmed to have an intelligence quotient (IQ) > 80 on the Draw-a-Man test^56^. To assess the psychiatric symptoms of participants, mothers were also asked to complete the BDI-II to evaluate their depressive symptoms^57^, and the AQ to evaluate both theirs and their children’s autistic symptoms^58,59^. No mothers had a BDI-II score above the cutoff. Subjects who had diagnoses of neurological illness, migraine, below average intelligence, or serious psychopathology were excluded from the study. Thus, seven children and their mothers were excluded from the final analysis; six children owing to excessive head motion and one owing to an AQ score higher than 25 points.

The protocol was approved by the Ethics Committee of the University of Fukui, Japan (Approval no. FU-20160120), and all procedures were conducted following the Declaration of Helsinki. All mothers and children provided written informed consent for participation in this study.

### Experimental procedure

The study included two sessions. In the first session, after explaining the experimental procedure and obtaining consent for participation, the mother and child were asked to alternately participate in brain scanning. While one underwent the brain scan, the other was asked to answer the various questionnaires described above. In the second session, the mother and child were engaged in three trials of table games using wooden toys (HABA Keep it Steady Game), and their interactions were recorded on video for 5 to 15 min in a quiet experimental room (with dimensions of 3.5 × 3.5 m) equipped with a table and chairs, with only the mother and child present. The position of the video camera was set so that the facial expressions of the mother and child could be confirmed. We instructed the mother to "in the parent–child game, please interact with your child in a natural way as usual." The recording of the parent–child interaction started when the mother received the toy from the experimenter.

### Brain-image acquisition and preprocessing

Image acquisition was performed using a 3-T MRI scanner (Signa PET/MR, ver. 26, GE Healthcare, Milwaukee, WI, USA) with a standard head coil (8-channnel HD Brain, GE Healthcare). Resting-state functional images were acquired with a T2*-weighted gradient-echo echo-planar imaging (EPI) sequence^60,61^. Each volume consisted of 40 slices, with a thickness of 3.5 mm and a 0.5-mm gap, to cover the entire brain. The time interval between two successive acquisitions of the same slice (repetition time; TR) was 2300 ms, with an echo time (TE) of 30 ms and a flip angle of 81°. The field of view was 192 × 192 mm, and the matrix size was 64 × 64, yielding volume dimensions of 3 × 3 mm. A total of 192 volumes were acquired for an imaging time of 7 min 42 s^60,61^. The participants were instructed to remain awake but to close their eyes and think of nothing in particular. Head movement was minimized by the placement of memory-foam pillows around the head. A T1-weighted anatomical dataset was obtained from each participant using a magnetization-prepared rapid acquisition gradient echo sequence (voxel size 1 × 1 × 1 mm, TE = 1.996 ms, TR = 6.38 ms, TI = 600 ms, flip angle = 11°, total scan time = 4 min 50 s)^60,61^.

### Resting-state fMRI data preprocessing

The rs-fMRI data were analyzed using Statistical Parametric Mapping (SPM) 12 (Wellcome Trust Centre for Neuroimaging, London, UK) with MATLAB R2016b (MathWorks, Natick, MA, USA) and the Data Processing Assistant for Rs-fMRI Software (DPARSF) toolbox^62^. First, the initial 10 volumes were discarded, and slice-timing correction was not applied, followed by spatial realignment of 186 volumes to the mean volume. The signal from each slice was realigned temporally to that obtained from the middle slice using “sinc” interpolation. To control for motion confounding in our data, we investigated the effects of head motion by computing the mean frame-to-frame root mean square motion and FD obtained during the realignment process^61^. Next, high-resolution T1 images were coregistered to functional images via a nonlinear image registration approach and segmented using a new segment algorithm with diffeomorphic anatomical registration through the exponentiated lie algebra technique^63^. Afterward, functional images were spatially normalized into the Montreal Neurological Institute template, resampled into a spatial resolution of 3 × 3 × 3 mm^3^, and spatially smoothened with a 6 mm full width at half-maximum Gaussian kernel^63^. Finally, the linear trend in the time series was removed, the non-neural noise in the time series was controlled, and several sources of spurious variance (e.g., the Friston 24-parameter model, white matter signals, and cerebrospinal fluid signals) were eliminated from the data through linear regression^60,61^.

### Behavioral assessment of mother–child interaction

To assess the degree of behavioral synchronization based on eye gaze in mother–child interactions, we extracted the number of gazes to the partner and the number of eye contacts from the video data while the participants were engaged in the game task. The numbers of one-sided gazes toward the partner (mother/child) (when the gaze was mutual, it was considered as eye contact) were counted. Then, as the total duration of engaging in the game task differed depending on the pair (5–15 min), the numbers of eye gazes and eye contacts were normalized by the duration and used as the indices for eye gaze.

In addition, the IRS was used to assess the quality of the mother–child interactions^24^. The IRS assesses the child’s social competence and the caregiver’s child-rearing competence based on a checklist of selected communication behaviors observed in a short period of time (approximately 5 min) in daily situations. The inter-observer reliability of the IRS was previously found to be 90%^24^. To rate the scale, the evaluator completes the checklist composed of 25 items focusing on the child’s behavior toward the caregiver (e.g., “Child vocalizes while looking at the task materials”) and 45 items focusing on the caregiver’s behavior (e.g., “Caregiver allows child to explore task material for at least five seconds before providing first task related instruction”). The observer then provides a score on a 5-point scale for the developmental level. The total internal consistency of the IRS was previously found to be excellent (α = 0.85–0.91)^64^. In this study, two observers (the first and second authors) who were trained by the original authors of the IRS assessed the videos, and the assessment was repeated until the concordance rate between the observers reached ≥ 80%.

### Statistical analysis

To investigate regionally specific correlates of resting-state brain function associated with the two gaze indices (number of eye gazes and of eye contacts), we performed multiple regression using SPM 12 and DPARSF. We executed fALFF analysis to determine the local resting-state fluctuations (RSFs) related to the two gaze indices for each mother and child. Each time series at each voxel as the fraction was calculated between the sum of amplitudes of the band ranging from 0.01 to 0.08 Hz, and subject-level voxel-wise fALFF maps were standardized into subject-level Z-score maps using the DPARSF toolbox^62^. The sex and age of the participants were included as covariates in the model due to their potential confounding effects. A threshold of cluster-level *P* ≤ 0.05 with a family-wise error correction for multiple comparisons and voxel-wise *P* ≤ 0.005 uncorrected was applied for the rs-fMRI data analysis. The anatomical localization of significant clusters was investigated with the Automated Anatomical Labeling and Broadman area atlases implemented in the Talairach Client software package^65^.

Demographics and clinical characteristics were tested using chi-squared and *t*-tests. Pearson's product-moment correlation coefficients were used to examine the relationships between the gaze indices and IRS score in mothers and children. The significance level was set to *P* < 0.05, and all statistical analyses were conducted using IBM SPSS Statistics and AMOS for Windows, version 20 (IBM Corp., Armonk, NY).
